# Percutaneous cryoablation for tuberous sclerosis-associated renal angiomyolipoma with neoadjuvant mTOR inhibition

**DOI:** 10.1186/1471-2490-14-77

**Published:** 2014-09-25

**Authors:** Thierry Krummel, Julien Garnon, Hervé Lang, Afshin Gangi, Thierry Hannedouche

**Affiliations:** 1 Department of Nephrology, University Hospital, Strasbourg, France; 2 Department of Interventional Radiology, University Hospital, Strasbourg, France; 3 Department of Urology, University Hospital, Strasbourg, France; 4 School of Medicine, University of Strasbourg, Strasbourg, France

**Keywords:** Tuberous sclerosis, Angiomyolipoma, Kidney, Sirolimus, Cryoablation

## Abstract

**Background:**

Renal angiomyolipomas (AMLs) are frequent in tuberous sclerosis and are responsible for a significant proportion of the morbidity in adulthood, mainly from bleeding complications, which are correlated to the size of the AMLs. We describe the case of a 19-year-old female with multiple bilateral renal angiomyolipomas.

**Case presentation:**

The renal AMLs measured up to 6 cm in size. She was first treated with a low dose of the mammalian target of rapamycin (mTOR) inhibitor sirolimus (up to 3 mg/day over a 12-month period) and following significant AML size reduction, percutaneous cryoablation was performed. No side-effects of either treatment were reported. At 12 months post-cryoablation, no recurrence of the AML was noted.

**Conclusion:**

This is the first report of this treatment strategy and the case study reveals that combining a low dose of an mTOR inhibitor with percutaneous cryoablation to treat small tumors mitigates the side-effects while providing a good clinical outcome. This therapeutic approach is a novel tool for the clinician involved in the management of patients with tuberous sclerosis.

## Background

Tuberous sclerosis complex (TSC) is an autosomal dominant genetic disease resulting from a *TSC1* gene (hamartin) or *TSC2* gene (tuberin) abnormality affecting one in 6000 births [1]. The mutation causes unregulated activation of the mammalian target of rapamycin (mTOR) pathway with development of benign tumors in multiple organs such as the kidneys, brain, liver, heart and skin [2].

Renal angiomyolipomas (AML) are common in patients with TSC, affecting nearly 80% of patients, and are responsible for a significant proportion of patient morbidity in adulthood [1]. The main risk is rupture of the AML with massive retroperitoneal bleeding [1]. The risk of hemorrhage is correlated to tumor size and the presence of aneurysms [3]. In addition, TSC can be complicated by kidney cysts in 30% of patients and carcinoma in 3% of patients [1]. Renal failure may occur as a consequence of repeated ablative treatments or as a consequence of cyst development, especially in patients with contiguous deletions in *TSC2* and *PKD1* (encodes polycystin-1) genes [4].

Classic therapeutic options for AML include partial nephrectomy, embolisation, radiofrequency and cryotherapy. In recent years, several studies have shown that mTOR inhibitors (mTORis) can significantly reduce the size of renal AML, with relatively tolerable side-effects [5,6]. The respective role of these treatments, however, remains elusive.

We report the case of a patient with TSC and a large renal AML treated by percutaneous cryotherapy after tumoral mass reduction induced by an mTORi. This is the first report of this novel treatment strategy.

## Case report

**Figure 1:**
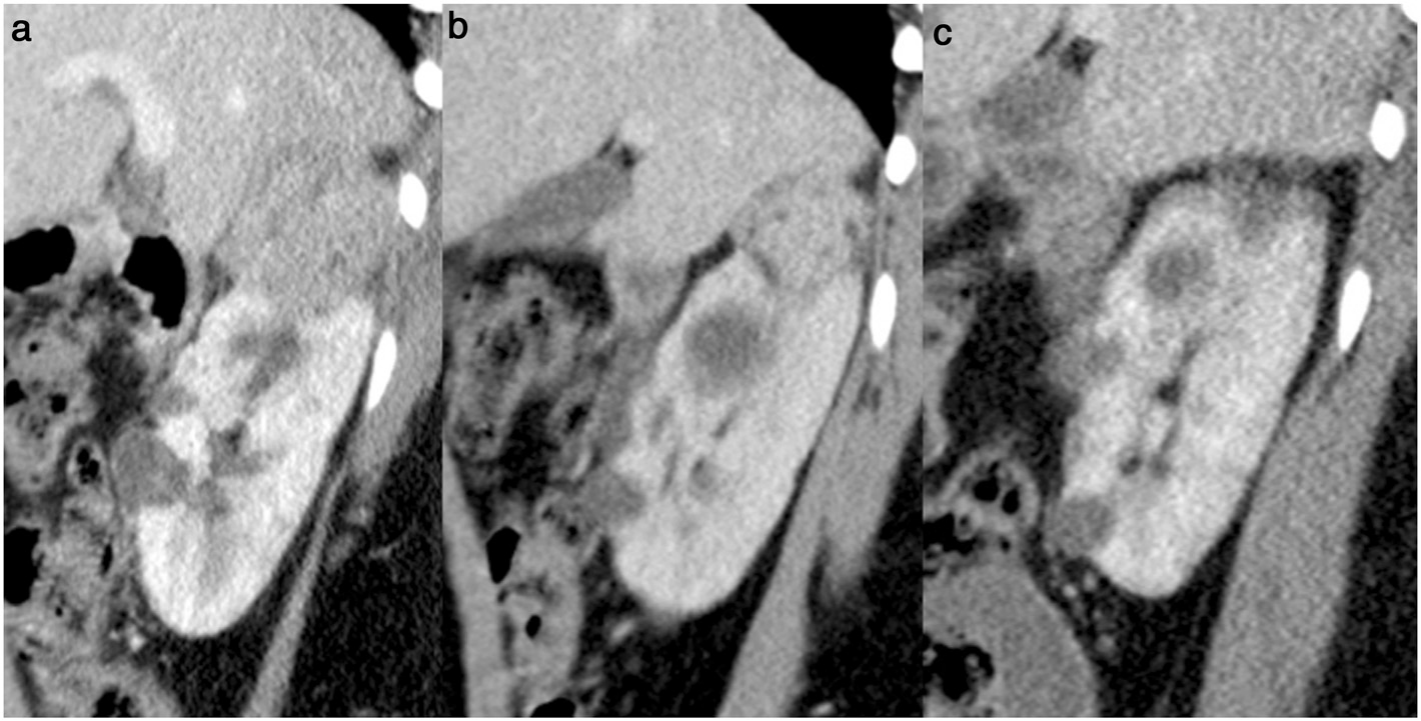
**Sagittal contrast enhanced (portal phase) computed tomography scan of the right kidney showing the angiomyolipoma of the upper pole. (a)** before treatment (left image); **(b)** after 12 months of treatment with sirolimus (middle image), showing a decrease in tumor size; and **(c)** one year after cryoablation (right image) showing devascularisation and shrinkage of the tumor, consistent with a complete ablation.

A 19-year-old woman was diagnosed with TSC after presenting with intractile epilepsy at 3 months of age. Seizure prophylaxis included carbamazepine, topiramate, lamotrigine, and a neurostimulator. She also presented with developmental delays, severe facial skin lesions asymptomatic lymphangioleiomyomatosis and obesity (body mass index 32.2 kg/m^2^). She had multiple renal AML, including one exophytic AML sized 6 x 5 x 4.5 cm at the upper pole of the right kidney (Figure 1), which required treatment.

An initial conservative approach consisted of progressively increasing doses of the mTORi sirolimus, up to 3 mg/day, over a 12 months period. After 6 months of maximal dose with sirolimus (plasma levels achieved: 2–3 ng/ml), the AML size was reduced to 4.5 x 4 x 3.5 cm (Figure 1). The reduction in size was substantial, although it was considered insufficient to reduce the bleeding risk and it was decided to proceed with. Percutaneous cryotherapy was selected as a treatment due to the available expertise at the treating institute and the favorable location of the AML.

**Figure 2:**
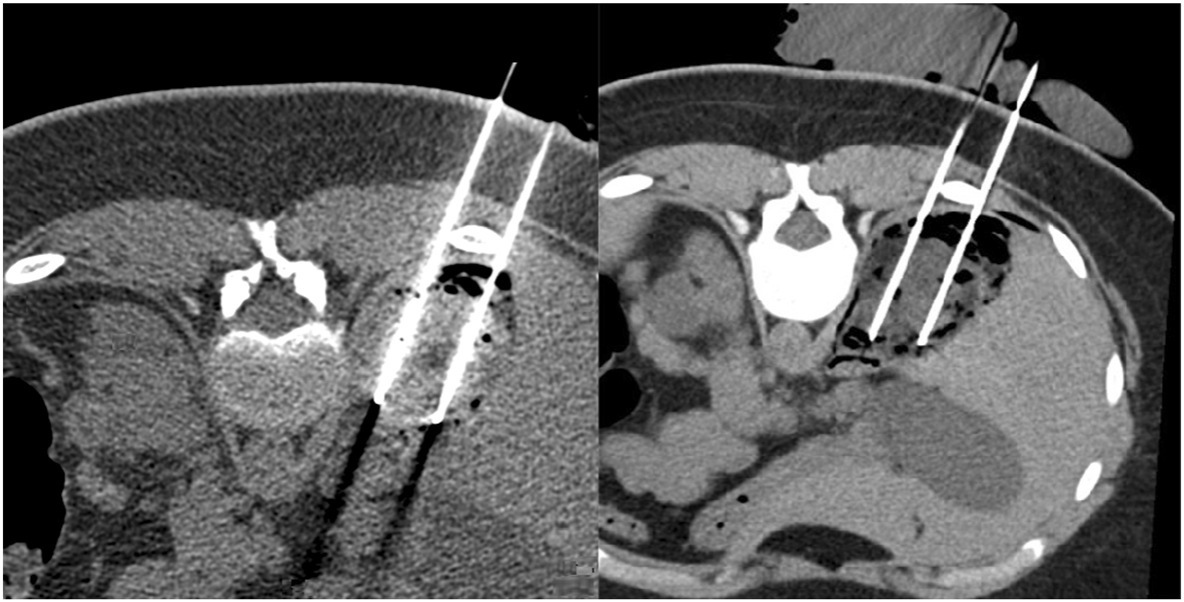
**Axial computed tomography (CT) slice showing 2 cryoprobes in the AML.** The bowel and the liver has been pneumodissected. Left image is before freezing and right image is after freezing.

Under general anesthesia, three cryoprobes were positioned in the AML from a posterior approach using computed tomography (CT) guidance (Figure 2). Cryoablation was performed with a standard protocol of 10 minutes freezing (−180°C) then ten minutes passive thawing and 10 minutes refreezing. The procedure was uneventful and complete coverage of the AML by the ice ball was achieved. Evaluation at 1 month confirmed the complete devascularisation of the AML. CT scans at 12 months post-cryoablation showed no sign of the treated AML (Figure 1), the other disseminated lesions within the two kidneys remained infracentimetric. Sirolimus treatment was maintained at the same level and subsequently replaced by everolimus, 5 mg/day, based on local drug agency approval. Everolimus residual plasma concentration remained within the range 2–3 ng/ml, which was below the recommended target of 5–10 ng/ml. However, after 12 months of treatment, the AML treated with cryotherapy showed no sign of recurrence and the size of the other AML lesions remained stable.

Renal function remained normal and stable throughout follow up, although a mild proteinuria (0.13 g/g of creatinine) occured. Hypercholesterolemia present pre-treatment increased slightly from 2.64 to 2.77 g/l, while triglycerides remained normal. Blood count was normal as well as the level of gammaglobulins. No side-effects of the treatment were reported.

## Discussion

The efficacy of mTORis in reducing the size of AML associated with TSC is now well documented. The decrease in size averages from 30 to 47% and has been shown to persist for up to 2 years [5,6]. However, the AML may recur upon discontinuation of the mTORi, warranting long-term treatment [6]. Although there is a likely benefit to this treatment, reduction in hemorrhagic risk with mTORi has not been formally demonstrated since bleeding events were infrequent in randomised trials [3]. The specific effect of the mTORi on the vascular component of the AML is not known but experimental data suggest that mTORis could partially inhibit the vascular pathogenesis of TSC [7].

In the current case study, the therapeutic response to sirolimus was a 25% reduction in size of the largest AML. This was considered insufficient to reduce the risk of bleeding and remaining therapeutic options were to increase mTORi dosage, perform a partial nephrectomy, or attempt embolisation or percutaneous ablative therapies.

In studies demonstrating the benefits of mTORis, initial target plasma levels were 1–6 ng/ml with potential titration to 9–15 ng/ml dependent on the response [6]. An increased dose was considered in this case study, however dose-dependent side-effects might have outweighed potential benefits [5,6]. Common side effects include stomatitis occurring in 30-85% of patients and mouth ulcers occurring in 16%, respiratory-type infections, wound healing defects, dyslipidemia, gastrointestinal disorders, and proteinuria also occur [8].

Partial nephrectomy is a definitive therapeutic option but is major surgery with a 5-23% rate of perioperative complications and a 16% conversion rate to radical nephrectomy [9,10]. Renal function can be acutely altered due to post-pedicle clamping ischemia or chronically due to nephron reduction. Berglund et al. showed a decrease in estimated glomerular filtration rate (eGFR) of 8 ml/min at 1 year post-surgery and chronic kidney disease prevalence of 14% in patients with normal kidney function preoperatively [9].

Staehler et al. recently published their results in three patients treated with 6 months of neoadjuvant sirolimus treatment, with plasma target level of 4–8 ng/ml, prior to partial nephrectomy [10]. A 38-95% preoperative reduction in AML size was observed and no major surgical complication or healing defect occurred. However, creatinine clearance decreased by 45 and 24 ml/min in two patients, respectively, and remained stable in the third patient.

Embolization is a common therapeutic option for TSC-associated AML with a primary 90-100% technical success rate; however, there is a 43% rate of recanalization and regrowth and a persisting bleeding risk warranting multiple embolizations [11,12]. The AML size reduction depends on the proportion of the fatty tissue, which is less sensitive to embolization [13]. Complications also include post-embolization syndrome secondary to infarction of the AML [14,15]. The effect on renal function appears minor if embolization is supra-selective and only affects the AML, but occasionally the feeding arteries of AML can be intrinsically involved with arteries to the normal parenchyma. Under these circumstances it is difficult to embolize without sacrificing normal renal tissue and this is a particular problem in TSC with multiples AML [16].

Percutaneous radiofrequency ablation is an *in situ* ablative technique used for many years with good results and no recurrence for up to 4 years [17,18]. However, the efficacy is relatively limited to the vascular component and the global size of the AML is only slightly reduced [17,18]. The particular efficacy on the vascular component explains why no bleeding has been reported in the small series published [17,18]. Complications are rare, being primarily neuralgia, even when large tumors (up to 32 cm max) are treated [17,18]. The effect of this technique on renal function has been negligible, with no significant observable decrease in eGFR at 2-year follow up [18,19].

Cryoablation of AML was first described using a laparoscopic approach with good results [20,21]. Subsequently, percutaneous cryoablation of AML has been reported in kidney cancer series with the advantage of being less invasive and the possibility of being repeated in case of residual tumors [22,23]. The only published series on renal AML included three patients who had small tumors of less than 2.5 cm [24]. Experience with percutaneous cryotherapy is much more substantial for renal cancers with many published series, mainly in patients with severe comorbidities contraindicating surgery. Complications of percutaneous cryoablation of kidney cancers occur in 5-15% of cases and are related to the patient's age, the size of the tumor and its proximity to the hilar vessels or collecting system [25,26].

Despite severe comorbidities and some minimal collateral normal tissue damage, renal function after cryotherapy has generally been well preserved with a decrease in eGFR of less than 10 ml/min/1.73 m^2^ after 24 months of follow-up [19]. The primary risk with this procedure is bleeding, which increases fourfold for tumors sized greater than 4 cm [25]. Due to this increased risk, current French recommendations on renal cancer treatment limit indications for percutaneous cryotherapy to tumors smaller than 4 cm [27]. Although, there is currently no published information to extrapolate these data to the treatment of kidney AML, similar caution is likely applicable.

In comparison with ablative and surgical treatments of TSC-associated AML, the use of mTORis remain poorly-defined. We suggest a strategy combining an mTORi with ablative treatment, in order to reduce AML size and allow safer radical cryotherapy. This strategy is also likely to avoid an unnecessarily high dosage of the mTORi and prevent medication related side-effects. Cryotherapy appears to have a long-term curative effect with minimal risk on AML smaller than 4 cm although its use in this indication and in combination with mTORi warrants further study in larger series.

## Consent

Written informed consent was obtained from the patient’s parents for publication of this case report and any accompanying images. A copy of the written consent is available for review by the Editor-in-Chief of this journal.

## Abbreviations

AML: Angiomyolipoma; eGFR: Estimated glomerular filtration rate.; mTOR: Mammalian target of rapamycin; mTORi: Mammalian target of rapamycin inhibitor; TSC: Tuberous sclerosis complex; PKD1: Polycystic kidney disease gene 1.

## Competing interests

The authors declare that they have no competing interests.

## Authors’ contributions

KT wrote the manuscript and performed the literature search. GJ performed the cryoablation and chose the images that illustrate the case. LH managed the patient in the Urology Department; he corrected the manuscript from a urological point of view. GA is the chief of the Interventional Radiology Department; he corrected the manuscript from a radiological point of view. HT is the chief of the Nephrology Department; he corrected the manuscript from a nephrology point of view. All authors read and approved the final manuscript.

## Pre-publication history

The pre-publication history for this paper can be accessed here:

http://www.biomedcentral.com/1471-2490/14/77/prepub
